# A periodic pattern generator for dental diversity

**DOI:** 10.1186/1741-7007-6-32

**Published:** 2008-07-14

**Authors:** Gareth J Fraser, Ryan F Bloomquist, J Todd Streelman

**Affiliations:** 1 School of Biology, Petit Institute of Bioengineering and Bioscience, Georgia Institute of Technology, Atlanta, GA 30332-0230, USA

## Abstract

**Background:**

Periodic patterning of iterative structures is a fundamental process during embryonic organization and development. Studies have shown how gene networks are employed to pattern butterfly eyespots, fly bristles and vertebrate epithelial appendages such as teeth, feathers, hair and mammary glands. Despite knowledge of how these features are organized, little is known about how diversity in periodic patterning is generated in nature. We address this problem through the molecular analysis of oral jaw dental diversity in Lake Malawi cichlids, where closely related species exhibit from 1 to 20 rows of teeth, with total teeth counts ranging from around 10 to 700.

**Results:**

We investigate the expression of conserved gene networks (involving *bmp2*, *bmp4*, *eda*, *edar*, *fgf8*, *pax9*, *pitx2*, *runx2*, *shh *and *wnt7b*) known to pattern iterative structures and teeth in other vertebrates. We show that spatiotemporal variation in expression pattern reflects adult morphological diversity among three closely related Malawi cichlid species. Combinatorial epithelial expression of *pitx2 *and *shh *appears to govern the competence both of initial tooth sites and future tooth rows. Epithelial *wnt7b *and mesenchymal *eda *are expressed in the inter-germ and inter-row regions, and likely regulate the spacing of these *shh*-positive units. Finally, we used chemical knockdown to demonstrate the fundamental role of hedgehog signalling and initial placode formation in the organization of the periodically patterned cichlid dental programme.

**Conclusion:**

Coordinated patterns of gene expression differ among Malawi species and prefigure the future-ordered distribution of functional teeth of specific size and spacing. This variation in gene expression among species occurs early in the developmental programme for dental patterning. These data show how a complex multi-rowed vertebrate dentition is organized and how developmental tinkering of conserved gene networks during iterative pattern formation can impact upon the evolution of trophic novelty.

## Background

Biology is replete with periodically patterned elements, from the sensory bristles of a fruit fly to the hair and teeth of mammals. Models of periodic patterning seek to explain the developmental origin of boundaries separating adjacent repetitive structures and the maintenance of cellular compartments once formed [1-8]. For example, the formation of feather tracts on the dorsal surface of chick embryos [9] and mammary (milk) lines on the ventral surface of embryonic mammals [10] serve to pre-pattern regions competent for the initiation of these structures. Similarly, a functionally equivalent field is established along the axis of the oral jaws in most vertebrates, competent to form tooth bud primordia [11-15]. In teleost fish this initial field is known as the primary odontogenic band (OB) [12,13,15] and in mammals it is termed the dental lamina [4,14]. This band or lamina sets the regionally restricted 'field' along the jaw axis from which tooth induction is triggered.

As with all periodically patterned systems, an initial 'field of competence' is set from a once-homogeneous cellular region, followed by the establishment of positional information throughout the restricted 'field' [16,17]. The initial field may be set up by a number of diffusing molecules such as morphogens that allow regionalization to occur, from which cellular differentiation responds along a gradient [5,18], probably by means of a reaction-diffusion-type mechanism [19,20]. Positional information determines cell differentiation, cellular compartmentalization and subsequent unit placode initiation, the first of which is imperative for iterative initiation of adjacent placodes via activator-inhibitor mechanisms [2]. Placode initiation is thought to be triggered by cellular accumulation (self-organization) over a given threshold that reacts to a number of positional cues within the competent field [1]. Within the placode itself, additional activators and inhibitors determine the boundaries of the placode unit and the spacing between units. Studies of periodically patterned systems such as the developing vertebrate dentition and developing chick feathers have led to the identification of a number of molecules that have been modelled as activators or inhibitors within the specific developing system [2,21,22]. In feather placode patterning, Shh and members of the Eda pathway have activator roles, while Bmp2 and Bmp4 are thought to act as inhibitors [2,23,24]. During mouse odontogenesis the same molecules are involved in patterning the molar cusps. Attempts have been made to model cusps according to activator-inhibitor patterning mechanisms; however, whether individual candidates can be classed as activators or inhibitors during tooth development is largely stage dependent [21,25-27].

Molecules involved in the establishment of vertebrate dentition have been well characterized from studies of the mouse [11,21,28]. A number of these molecules are known to have detrimental effects on the murine dentition when removed/inhibited from the dental network early in tooth development; *Shh *[29,30], *Pitx2 *[31] and *Pax9 *[32] are among those with severe dental phenotypes [33]. For example, inhibition of *Shh *in mandibular explants during the transition of dental competence to initiation (E10.5) leads to tooth arrest at the bud stage [29,30]. Thus, it is clear that this gene is essential for the correct establishment of the global dental programme. However, these studies are specific to the mouse experimental model, which develops a single set of teeth with no replacements. We therefore know nothing of the resulting phenotypes when modifications occur to these networks, for example the hedgehog pathway, in vertebrates with numerous functional tooth rows and continuous replacement cycles.

**Figure 1:**
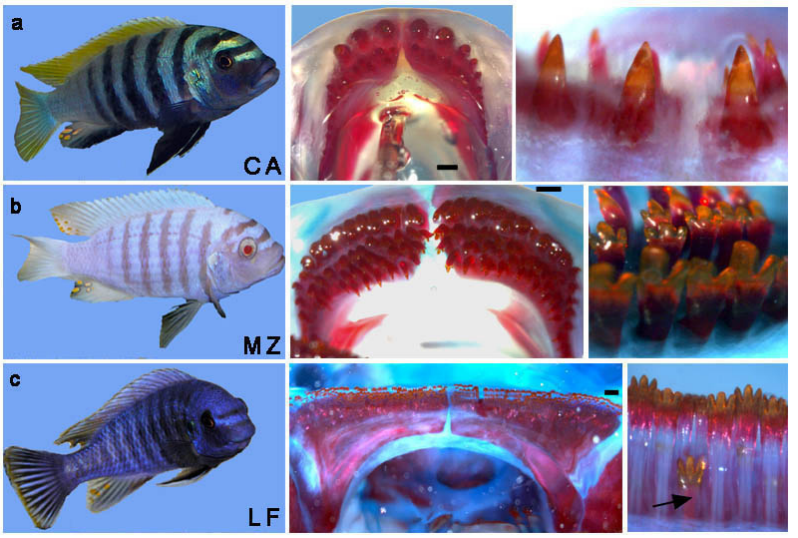
**Nature's experiment in dental diversity among Lake Malawi cichlids**. (a) *Cynotilapia afra *(CA) has a crescent-shaped jaw with two rows of widely spaced unicuspid teeth. (b) *Metriaclima zebra *(MZ) exhibits an intermediate jaw shape with five to six rows of teeth; a first row of bicuspid teeth is followed by several lingual rows of tricuspids. (c) *Labeotropheus fuelleborni *(LF) has a square-shaped jaw, lined with 10 or more rows of tightly packed tricuspid teeth. The arrow marks a replacement tooth in LF. Jaws were prepared with alizarin red, which stains the bone matrix and dentine. Scale bars in the lower jaw images are 500 μm. Lower jaw images are dorsal view.

The morphogenesis of teeth, like that of other periodically patterned vertebrate organs (for example, hair, mammary glands, feathers), is regulated both by sequential and reciprocal molecular interactions between two adjacent cell layers, the epithelium and the directly underlying mesenchyme [11,28]. During early stages, these distinct organs share a number of features and express a familiar suite of genes with common roles [28,34]. Many studies have attempted to identify the morphodynamic control of iterative organization and how such patterning mechanisms change during development to generate evolutionary novelty [6,7,21,35-37]. We sought to characterize the expression of a set of these molecules in the dentitions of Lake Malawi cichlids to tackle an unanswered and fundamental biological question: how is the diversity of periodically patterned elements generated in nature? Malawi cichlids are exemplars of natural craniofacial diversity. In essence, natural selection has conducted an experiment in micro-evolutionary diversification, and we want to know how development works to produce variation in phenotype [37]. The range of dental variety in Malawi is tremendous given a common ancestor in the last 500,000 to 1 million years [26]; species possess about 10 teeth in a single row (per jaw), or as many as 700 teeth in up to 20 rows. Species differ in tooth size, spacing and shape in coordinated fashion [26,36]. We focus on three closely related Lake Malawi cichlids with alternative dental phenotypes (Figure 1): *Cynotilapia afra *(CA, Figure 1a), a unicuspid species with two tooth rows of large, widely spaced teeth; *Metriaclima zebra *(MZ, Figure 1b), a bicuspid and tricuspid species with five or six tooth rows of intermediately sized and spaced teeth; and *Labeotropheus fuelleborni *(LF, Figure 1c), a uniformly tricuspid species with 10 or more tooth rows of small, tightly packed teeth [26,36]. Previously, we have used these species to identify the chromosomal basis of divergent tooth shapes among species [26,36]. Here we address a different question: we ask how conserved gene networks are deployed to influence the diversity in the size, number, spacing within rows and number of rows of teeth.

## Results and Discussion

### Variation in developmental gene networks prefigures differences in adult cichlid dentitions

We cloned cichlid orthologues of genes required during oral epithelial organization and tooth germ initiation (*bmp2*, *fgf8*, *pitx2*, *shh*) [11,38] as well as mesenchymal markers (*bmp2*, *bmp4 *[36], *pax9*, *runx2*) involved in reciprocal signalling to the epithelium [11]. Teleost tooth development has been well characterized in the zebrafish and thus our nomenclature for the early stages of tooth development will follow that model. Two stages of early odontogenesis are relevant: the thickened epithelium stage and the bell-shaped epithelium stage [39-41]. Developing teeth beyond this point will be referred to as tooth germs, spanning the progression of the tooth from a bell-shaped unit to various stages of functional maturity, characterized by cytodifferentiation.

**Figure 2:**
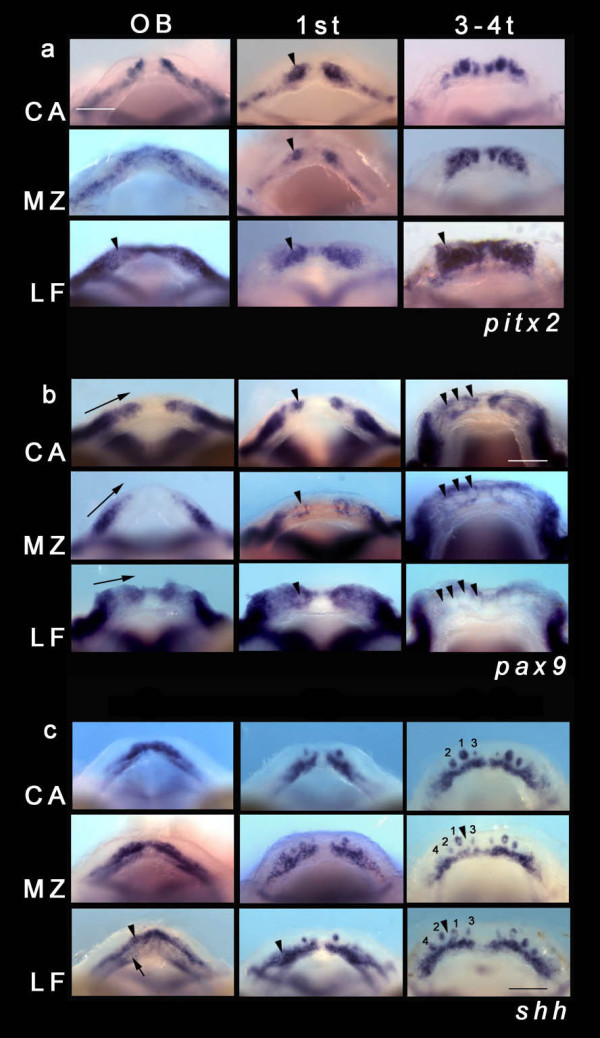
**The developmental program of Malawi cichlid dentitions**. (a) Expression of *pitx2 *in the lower jaw (dorsal view) of three Malawi cichlid species, *Cynotilapia afra *(CA), *Metriaclima zebra *(MZ), *Labeotropheus fuelleborni *(LF), during tooth initiation and development. OB, odontogenic band (around 4 days post-fertilization (dpf)); 1^st^, first tooth to initiate (around 5 dpf); 3–4 t, 3–4-teeth stage (around 6–7 dpf). Black arrowhead in LF OB shows the extent of the initial field of odontogenic competence prior to tooth germ initiation. Black arrowheads in CA, MZ, LF 1^st ^indicate the formation of the first tooth, note the extensive region of competence in LF, later marking competent epithelium reflecting the future distribution of teeth (arrowhead, 3–4-teeth stage; refer to Figure 1c). Scale bar 100 μm. All panels are to the same scale. (b) Expression of *pax9 *in the lower jaw (dorsal view) of three Malawi cichlid species CA, MZ, LF, during tooth initiation and development. OB, 1^st^, and 3–4 t as in (a). Black arrows in OB for CA, MZ and LF mark the extent of the odontogenic field of expression within mesenchymal cells along the mesiodistal jaw axis; the point of the arrow indicates the position along this axis where the first tooth will initiate. Black arrowheads in 1^st ^for CA, MZ and LF point to up-regulated expression in the mesenchyme surrounding the thickening dental epithelium. Black arrowheads in CA, MZ and LF 3–4 t, show developing teeth with *pax9 *expression in the dental mesenchyme surrounding the epithelial tooth germ, and not within the dental papilla. Scale bar 100 μm. All panels are to the same scale. (c) Expression of *shh *in the lower jaw (dorsal view) of three Malawi cichlid species CA, MZ, LF, during tooth initiation and development. OB, 1^st^, and 3–4 t as in (a). Black arrowhead in LF OB marks the onset of tooth initiation within the primary OB (note the asymmetry of dental initiation, left half of the dentary initiating first); tooth initiation will occur within this restricted band although the area of dental competence is extended lingually (black arrow). Black arrowhead in LF 1^st ^shows the extended OB. MZ and LF 3–4 t shows the order of initiation and spacing of the first teeth. Numbers refer to the order of appearance. Black arrowhead marks the future position of tooth 5, which is placed differently in MZ versus LF. Scale bar 100 μm. All panels are to the same scale.

**Figure 3:**
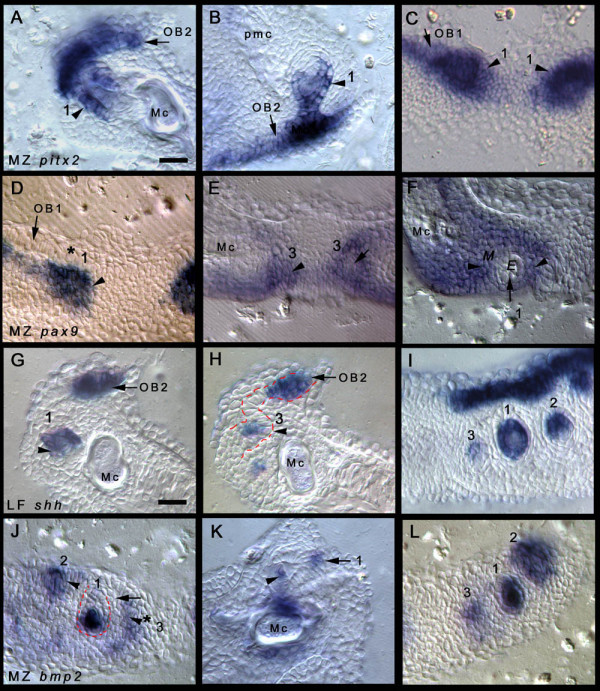
**Thin sections of the developmental program of Malawi cichlid dentitions**. (A)-(C) *Metriaclima zebra *(MZ) *pitx2 *expression within the oral and dental epithelium. (A), (B) 3–4-teeth stage, 7 dpf; (C) first-tooth stage. (A) Sagittal section (dentary), expression in the dental epithelium (tooth number 1 in the series, inner dental epithelium (IDE) and outer dental epithelium (ODE); black arrowhead). Continued dental competence for the next row (odontogenic band (OB) 2) is shown by expression in the lingual extent of the oral epithelium (arrow, OB2). (B) Sagittal section (upper jaw); *pitx2 *is expressed in the IDE and ODE (arrowhead, tooth number 1 in the series). Expression within the basal epithelial cells denoting the competent oral epithelium for future tooth initiation (OB2; arrow). (C) Coronal section (dentary) initial thickened epithelia of the first-tooth germs (1, thickened dental epithelium; black arrowheads) labelled by *pitx2*. The competent field (OB1) continues laterally from which new adjacent tooth germs will develop (arrow). (D)-(F) MZ *pax9 *in the dental/oral mesenchyme underlying the thickened epithelium (D) (thickening stage of the first tooth) and surrounding the developing dental units (E), (F) (3–4-teeth stage, 7 dpf). (D) *pax9 *(arrowhead) in mesenchyme (asterisk, dental epithelium, first tooth). Continued lateral expression in mesenchyme underlies the odontogenic band of the first row (OB1, arrow). (E), (F) Oblique coronal sections (dentary) of MZ. (E) *pax9 *expression is observed within the dental mesenchyme (arrowhead) of tooth 3 in the series (medial, on each half of the dentary) and surrounding the developing epithelial tooth germ (arrow) within the mesenchyme and in (F); for orientation, the thickened epithelium is forming down into the page; epithelium (E), mesenchyme (M, arrowheads), tooth 1 (arrow, 1, developing down into the page). (G)-(I) LF *shh *expression in the dental epithelium (3–4-teeth stage, 6 dpf). (G) Sagittal section (dentary), expression in the IDE of the first tooth in the series (1, black arrowhead). Black arrow, competent OB already marking the presumptive second row (OB2). (H) Epithelial thickening stage of tooth 3 in the series (3), sagittal section *shh *in the thickened dental epithelium (black arrowhead). The red dashed line demarcates the epithelium joining the second OB (OB2, black arrow) with the tooth germ. (I) Coronal section (dentary) showing the first three tooth germs in the series (1–3 in order of development) *shh *is restricted to the IDE of tooth 1, the epithelial germ in tooth 2 and the thickened epithelium in tooth 3. Directly above the tooth germs (in this plane of section) is the second OB for the initiation of subsequent teeth. (J)-(L) MZ *bmp2 *in both the dental epithelium and dental papilla (mesenchyme) (3–4-teeth stage, 7 dpf). (J) Oblique coronal section (dentary) expression within thickened epithelium for teeth 2 and 3 in the series (epithelium; arrowhead). More mature developing tooth (number 1, red dashed line) showing dental papillary expression (mesenchyme). The region between the 3 tooth units of varying stages, the ZOI, contains no *bmp2 *expression (arrow). Dental epithelium of tooth 3 initiating epithelial thickening stage, (arrowhead with asterisk, 3) well spaced from the neighbouring germ. (K) Sagittal section (dentary) the maturing (cytodifferentiation) first tooth (1) showing *bmp2 *in the dental papilla (mesenchyme; arrowhead) and simultaneous expression in IDE cells at the tip of the tooth (arrow), equivalent to the primary enamel knot in mammals. (L) Coronal section (dentary); order of initiation of the first three teeth (1–3) showing the variety of *bmp2 *expression. First tooth (1) shows dental papillary expression, second and third tooth to initiate (2, 3) show epithelial expression, more so in (2) as developing tooth germ is present. All images are to the same scale; scale bar in (A) is 20 μm. Sections cut to a thickness of 25 μm. E, epithelium; M, mesenchyme; Mc, Meckel's cartilage; pmc, premaxillary cartilage.

The transcription factor *pitx2*, described as a putative odontogenic-commissioning gene [12,13,15], has a broad expression pattern that encompasses both the developing tooth unit and the inter-tooth region, marking the extent of the dental-competent oral epithelium, including regions of future tooth rows (Figures 2a and 3A–C). *pitx2 *is one of the earliest dental epithelial markers (Figure 3C) with expression in the thickened dental epithelium (Figure 3C) and both the inner dental epithelium (IDE) and outer dental epithelium (ODE) of the maturing tooth (Figure 3A and 3B). Interestingly, the early pattern of *pitx2 *expression differs across the three species prior to and during morphogenesis of the first tooth, and reflects the future organization of these distinct dentitions (Figure 2a). *L. fuelleborni *shows the greatest region of dental competence (expression of *pitx2*), consistent with the later elaboration of teeth and tooth rows (Figures 1c and 2a). Future tooth rows also show expression of *pitx2*, labelling the lingual progression of the subsequent OB (Figure 3A and 3B). Similar to studies in other fishes [38,41], we found that *fgf8 *expression is not associated with initiating tooth germs in Malawi cichlids (not shown).

*pax9*, one of the earliest mesenchymal markers of odontogenesis in the mouse, is either absent from or weakly expressed in the dentitions of zebrafish and Mexican tetra [38]. By contrast, in Malawi cichlids *pax9 *is expressed initially in oral mesenchymal cells as a dental field along the mesiodistal jaw axis (Figure 2b, OB stage, and Figure 3D), then it is strongly up-regulated in the underlying mesenchyme at the epithelial thickening stage of the first tooth (Figure 2b, first-tooth stage, and Figure 3D). Expression of *pax9 *is then restricted to cells of the dental mesenchyme enveloping the tooth during morphogenesis and is absent from the cells of the dental papilla (Figure 2b, 3–4-teeth stage, and Figure 3E and 3F). The expression of *runx2 *essentially replicates that of *pax9 *for the stages examined (data not shown).

**Figure 4:**
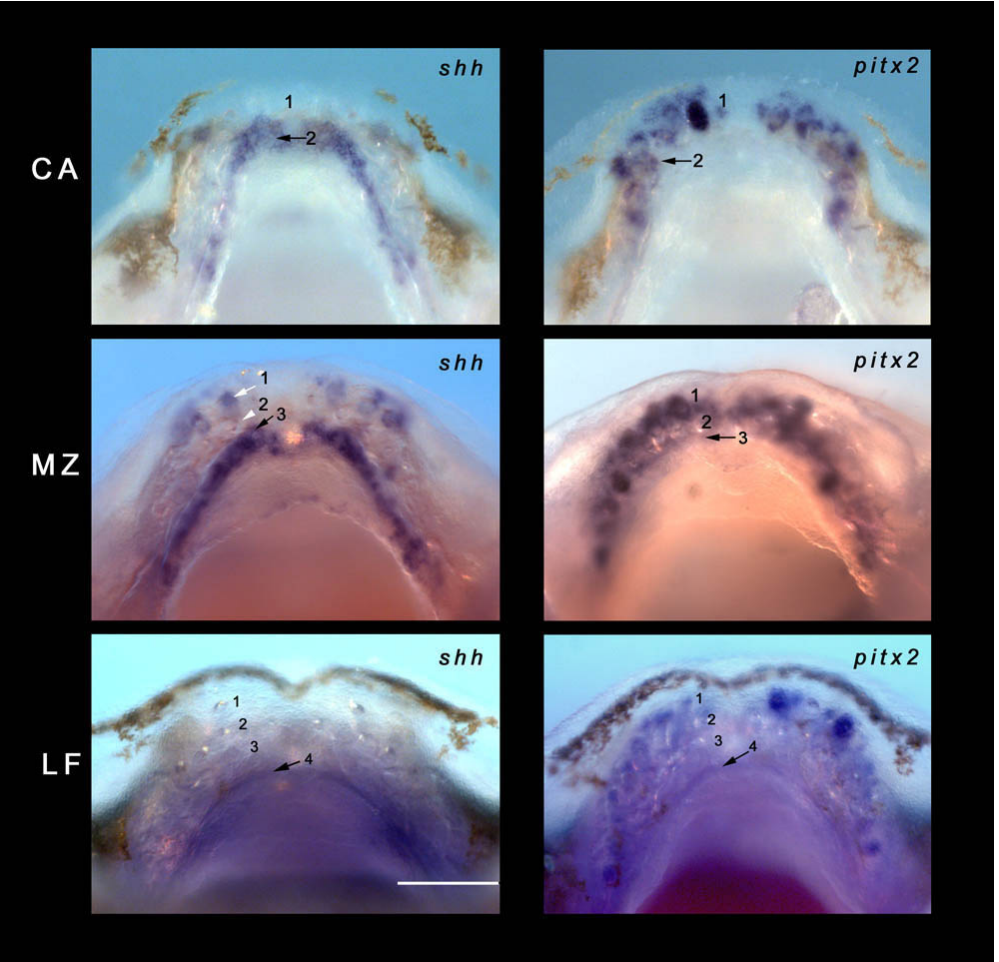
**Patterning multiple tooth rows in Malawi cichlids**. *shh *and *pitx2 *are co-expressed in an odontogenic band (OB) for every new tooth row. *shh *is expressed in a second OB in *Cynotilapia afra *(CA) (arrow, 2) (around 12 days post-fertilization (dpf)); the second row has started the process of initiation (*pitx2 *CA arrow, 2). *pitx2 *is absent from the lingual extent of the jaw margin and we propose that this lack of *pitx2 *(and therefore combinatorial expression of both *pitx2 *and *shh*) is in part responsible for the lack of additional rows (3, 4 and so on) in CA. *Metriaclima zebra *(around 12 dpf) initiates a third row and *Labeotropheus fuelleborni *(around 12 dpf) row 4, concomitant with OBs labelled by both *shh *and *pitx2*. Expression is also visible in replacement teeth in the first rows. Scale bar 100 μm.

Expression of *shh *is up-regulated from the primary OB into the individual tooth germs (Figure 2c and 3G–I). *shh *continues to be expressed during tooth morphogenesis, marking the bell-shaped dental epithelium and later the IDE of the tooth during cytodifferentiation (Figure 3G–I). Notably, *shh *is never present in regions between or around teeth, called the zone of inhibition (ZOI) [15-17]. In Malawi cichlids *shh *expression continues to label OBs, marking subsequent initiation of more lingual tooth rows, one at a time (Figures 2c and 3G–I). *C. afra *does not develop a third OB (nor a third row), while *M. zebra *and *L. fuelleborni *initiate an OB for each future tooth row (see Figure 4). *bmp2 *is co-expressed in the competent epithelial OB with *shh *and *pitx2*. From the initial epithelial OB, *bmp2 *expression is up-regulated in the cells of the contorted bell-shaped epithelial germ and continues to be expressed during differentiation (Figure 3L) before becoming localized to cells of the mesenchymal dental papilla (Figure 3J–L). In addition, *bmp2 *is restricted to epithelial cells at the developing tooth tip (Figure 3K), which will differentiate to ameloblasts, partially responsible for the secretion of enameloid, the first mineralized tissue of the teleost tooth [42]. These are an equivalent set of cells to the mammalian 'enamel knot'. *bmp4 *is expressed initially in the mesenchymal field along the mesiodistal axis prior to tooth germ initiation, much like *pax9 *and at the 3–4-teeth stage *bmp4 *is restricted to the dental papilla (data not shown [36]).

These data, in conjunction with data reported earlier for *bmp4 *[36], suggest that early patterns of gene expression differentiate among Malawi cichlid dentition types. *L. fuelleborni*, *M. zebra *and *C. afra *exhibit marked variation in the field of odontogenic competence both in the epithelium (*pitx2, shh*) and mesenchyme (*pax9*), in the spacing (*bmp2*, *bmp4*, *pax9*, *runx2*, *shh*) and in the size (*bmp4*, *pax9*, *runx2*, *shh*) of the initial tooth germs. These differences are readily quantifiable (Table 1), arise before teeth acquire their functional adult shape, and correspond directly to the size and spacing observed in the adult dentitions [26]. For example, *C. afra *embryos have the largest initial dental germs and surrounding ZOI (Table 1) while adults have the largest, fewest teeth (mean ± SD of 1.3 ± 0.20 per millimetre of jaw width) with the greatest inter-unit spacing compared with the other two species, *M. zebra *(3.3 ± 0.52) and *L. fuelleborni *(4.8 ± 0.74) [26]. The measurements obtained from *shh *expression in Table 1 highlight an early developmental origin of dental diversity among these three species. Streelman and Albertson noted a similar pattern from *bmp4 *expression, with a comparable range of cichlid species [36]. While some measurements shown here seem counterintuitive (for example, Table 1b, columns 3 and 4), it is because species also differ in more subtle aspects of tooth initiation. *C. afra *never initiates a tooth between the first three teeth to develop; *M. zebra *has an initiation order that places tooth 5 between teeth 1 and 3, whereas *L. fuelleborni *initiates tooth 5 between teeth 1 and 2 (Figure 2c). These early differences in tooth size, spacing and organization are perhaps surprising because cichlids (and all teleost fish) continuously replace their teeth; therefore, we might have expected inter-specific variety to develop gradually, over multiple rounds of tooth replacement, from a common dental 'ground' state, as is the case for tooth shape [26].

**Table 1:** Size and spacing of tooth germs in three species of Malawi cichlids

(a)								
**Species**	**Diameter of first tooth**	**ZOI including first tooth**	**ZOI medial to first tooth**	**ZOI distal to first tooth**	**ZOI from First tooth to second OB**	**MD length of OB**	**Width of OB at level of first tooth**	**Area (μm^2^) of first tooth**

*CA*	22.91 ± 0.839	51.80 ± 1.298	18.67 ± 0.344	17.99 ± 0.482	8.80 ± 0.56	110.72 ± 7.337	22.21 ± 1.135	528.08 ± 31.348
*MZ*	17.62 ± 0.149	44.05 ± 0.898	16.50 ± 0.213	12.87 ± 0.322	6.84 ± 0.084	116.62 ± 5.013	23.02 ± 0.382	384.06 ± 4.754
*LF*	14.87 ± 0.349	39.9 ± 1.697	11.95 ± 0.285	15.24 ± 0.392	7.28 ± 0.545	120.91 ± 3.834	19.41 ± 0.782	268.47 ± 8.084

(b)								
	
**Species**	**Diameter of first tooth**	**Inter-germ space between first and next medial tooth**	**Inter-germ space between first and next distal tooth**	**ZOI from First tooth to second OB**	**MD length of second row OB**	**Area (μm^2^) of first tooth**		
		
*CA*	25.68 ± 0.359	16.64 ± 0.128	14.02 ± 0.096	8.29 ± 0.266	140.02 ± 3.669	684.57 ± 19.248		
*MZ*	18.19 ± 0.231	19.63 ± 0.222	11.96 ± 0.318	10.18 ± 0.434	138.47 ± 2.897	435.09 ± 19.98		
*LF*	18.37 ± 0.437	13.06 ± 1.112	20.33 ± 1.065	8.02 ± 0.295	168.39 ± 5.432	404.15 ± 23.182		

(a) We report mean values (three to five individuals) of measurements taken from the expression of *shh *at the first-tooth stage in the three cichlid species: CA, *Cynotilapia afra*; MZ, *Metriaclima zebra*; LF, *Labeotropheus fuelleborni*. MD, mesiodistal; OB, odontogenic band; ZOI, zone of inhibition. (b) Mean values (three to five individuals) from the 3–4-teeth stage. All values are in micrometres ± SE.

### Organizing the periodic pattern with molecular 'spacers'

**Figure 5:**
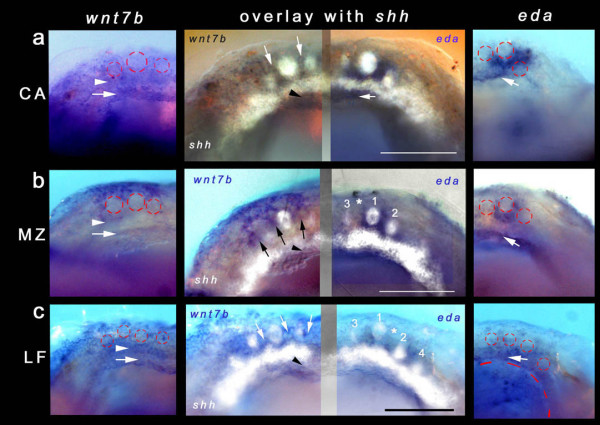
**Organizing Malawi cichlid dentitions with the 'spacer' gene *wnt7b *and *ectodysplasin***. Left panel: *wnt7b *is expressed in the zone of inhibition (ZOI) and inter-germ/inter-row spaces in (a) *Cynotilapia afra *(CA), (b) *Metriaclima zebra *(MZ) and (c) *Labeotropheus fuelleborni *(LF). Red circles indicate *shh*-positive tooth germs; arrowheads point to expression associated with initiating first-row teeth; arrows mark a lingual band of expression demarcating the *shh *odontogenic band (OB) of the second tooth row. Right panel: *eda *expression is up-regulated lingually in association with the mesenchyme of the first teeth (red circles) of (a) CA, (b) MZ and (c) LF. Expression is strongest in CA and most diffuse in LF. Arrows mark a second band of expression (CA, MZ) or continued expression (LF) lingually. Middle panel: Composite jaws showing the expression of *wnt7b *(left) and *eda *(right) in overlay with *shh *(false-colour, white). Arrows and arrowheads indicate expression in the ZOI of first-row teeth and in association with the second-row OB. For MZ and LF, tooth positions are numbered in order of initiation and asterisks mark the position of tooth number 5. All images from the 3–4-teeth stage; all specimens were stage-matched based on external structures, that is, pectoral and caudal fin development and eye development and maturity (3–4-teeth stage for CA and LF was 6 days post-fertilization (dpf) and for MZ it was 7 dpf). Overlay images from different individuals may show artefacts from slight differences in alignment. Scale bars for the middle panel are 100 μm.

Given the set of molecules localized to the first tooth germs (notably *shh*, but also *pitx2 *and *bmp2*), we hypothesized that other factors expressed within the ZOI surrounding these germs might guide the size and spacing of early cichlid tooth units. We therefore analyzed the expression of genes involved in a putative spacing mechanism. We cloned three genes, *eda*, *edar *and *wnt7b*, with antagonistic effects on *shh *in the initiation of mouse teeth, mouse hair follicles and chick feathers [9,30,43-50]. *edar *(data not shown) is expressed within the germs themselves in a pattern similar to *shh *(Figures 2c and 3G–I). We observed the expression of both *wnt7b *and *eda *surrounding the initial *shh*-positive tooth germs (Figure 5a–c) within the ZOI/inter-germ regions across the three species.

**Figure 6:**
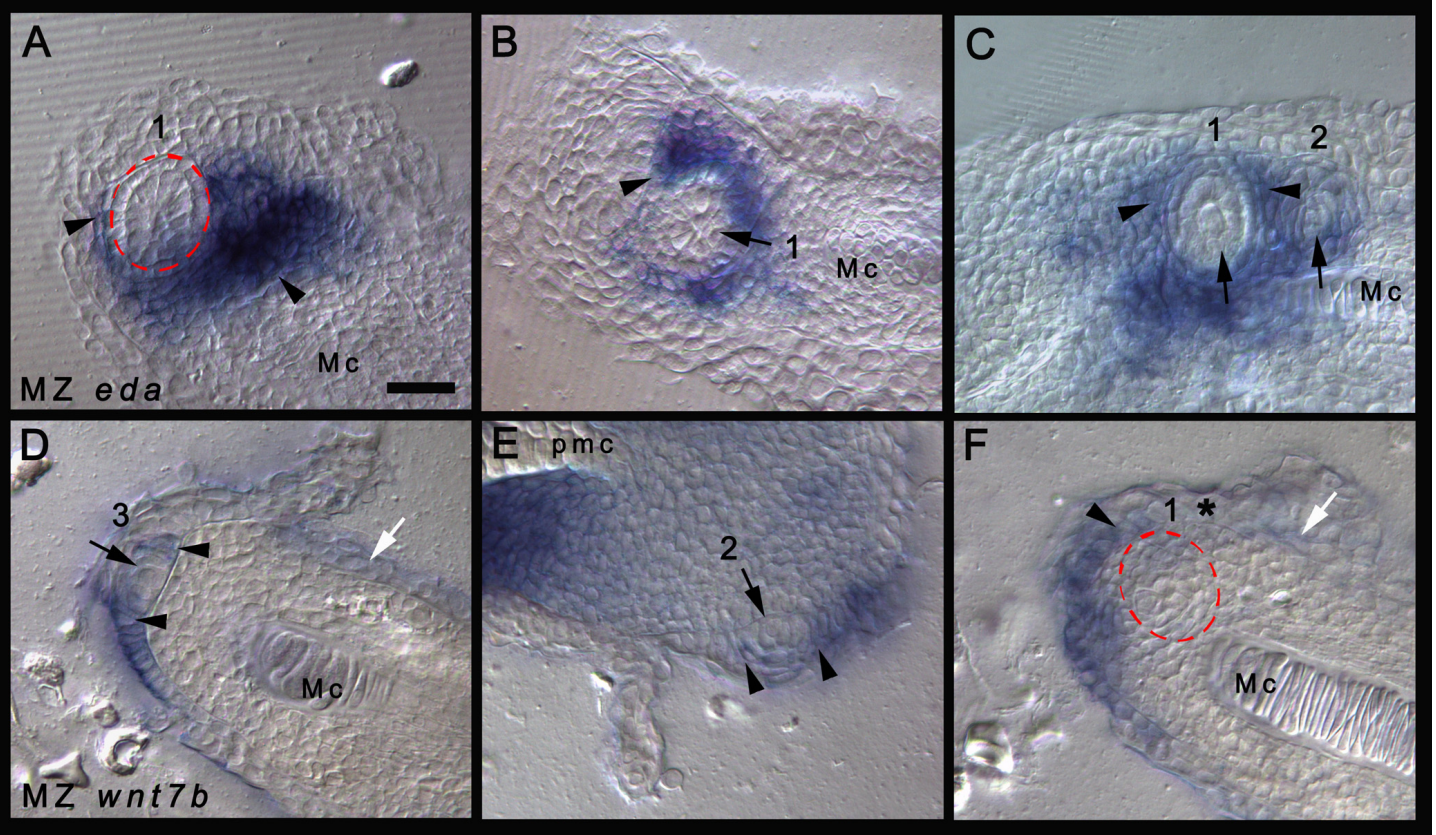
**Regulating tooth size and spacing**. Thin sections of *Metriaclima zebra *(7 days post-fertilization; 3–4-teeth stage). (A)-(C) *eda *expression within the mesenchyme (black arrowheads) surrounding the tooth germs (red dashed circle or black arrows; numbers represent the order of appearance for the tooth shown). Note the expression of *eda *is restricted to the mesenchyme and is not present in the epithelium. (D)-(F) The expression of *wnt7b *is restricted to the epithelium (D), (E) (black arrowheads) either side of the thickened dental epithelium (black arrow) and in inter-row space (white arrow). (F) A dentary tooth germ (red dashed circle, tooth 1) devoid of *wnt7b *expression. Expression is restricted to the non-dental epithelium (black arrowhead); numbers 1–3 refers to the order of tooth appearance in the series; asterisk denotes the dental epithelium that will express *shh *(see Figure 3G–I) and lacks *wnt7b*. *wnt7b *expression is present lingually restricting the second odontogenic band (white arrow, (D) and (F); see Figure 5). Sections cut at a thickness of 25 μm. Scale bar in (A) is 20 μm; all panels are to the same scale. Mc, Meckel's cartilage; pmc, premaxillary cartilage.

*eda *is expressed locally and strongly surrounding the first-tooth germ (expressing *shh *and *edar*) in all three species (first-tooth stage data not shown). By the 3–4-teeth stage, *eda *remains expressed in the mesenchyme locally and heavily at the lingual margin of the first tooth in *C. afra *and *M. zebra *(Figures 5a and 5b and 6A–C), but its lingual expression is broader and more diffuse in *L. fuelleborni *(Figure 5c). Notably, there appears to be a lateral bias in the expression of *eda *in both *L. fuelleborni *and *M. zebra*, which may reflect the influence of (or may influence) the initiation of tooth 5 between existing germs 1 and 2 or 1 and 3, respectively. An apparent *eda*-negative region exists in both *L. fuelleborni *and *M. zebra *where the fifth tooth in the series will appear, a different position in each species (Figure 5, middle column). *C. afra *lacks tooth initiation between these first three positions, an arrangement that continues into the adult dentition.*wnt7b *expression coincides with the ZOI surrounding the first teeth in all species (Figures 5 and 6D–F); this is best illustrated comparatively with image overlays with *shh *as depicted in Figure 5a–c.

Remarkably, these genes seem also to be employed in the initiation and spacing of future tooth rows, an iterated expression pattern similar to tooth germ organization within each row. *shh *labels each OB for subsequent tooth rows (Figures 2c and 4); *eda *and *wnt7b *are expressed between the first tooth row and the OB of the second (Figures 5 and 6). Specifically, *eda *expression partly overlaps that of *shh *in the lingual OB, while *wnt7b *is expressed either side of *shh*. Thus, eda from the enveloping mesenchyme (Figure 6A–C) may induce and maintain *shh *expression in tooth germs as well as in future tooth rows, and planar epithelial wnt7b (Figure 6D–F) may inhibit dental competence in these regions, similar to the role of these molecules in other systems [9,30,44-48].

### Hedgehog signalling is required for initiation of periodic dental patterning

**Figure 7:**
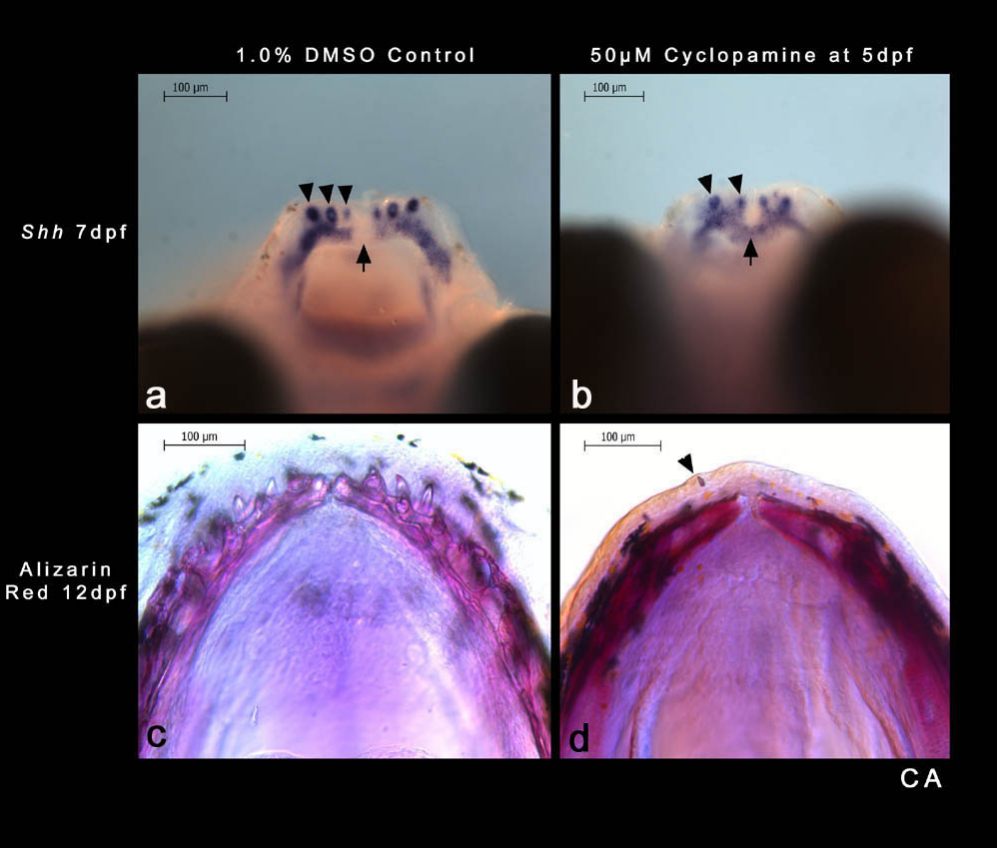
**The hedgehog pathway is essential for dental periodic patterning in Malawi cichlids**. Control (1% DMSO) treated (a) and cyclopamine treated (b) *Cynotilapia afra *embryos (7 days post-fertilization (dpf)) showing *shh *expression in the lower jaw dentition (arrowheads). Cyclopamine (and DMSO) was administered to *C. afra *embryos at 5 dpf for 24 hours; embryos continued to develop for a further 24 hours under standard conditions and were fixed at 7 dpf. Weaker levels of *shh *expression in cyclopamine-treated fish along with a disturbed initial pattern of tooth germs and second odontogenic band (b) compared with the 1% DMSO control (a). Note three tooth germs on each half of the dentary in the control (a) compared with two tooth germs on each half of the dentary in embryos treated with cyclopamine (b) (arrowheads). *C. afra *that were treated for 24 hours at 5 dpf (as above) continued to develop for a further 6 days (12 dpf) to stages where teeth of the first row are expected to erupt and teeth should be developing in the second tooth row (see Figures 4 and 8). Compared with the 1% DMSO control (c) the cyclopamine-treated *C. afra *(d) failed to develop a dentition; a single tooth shard (arrowhead) is seen unattached within the epithelium above the ossified lower jaw (alizarin red preparation).

**Figure 8:**
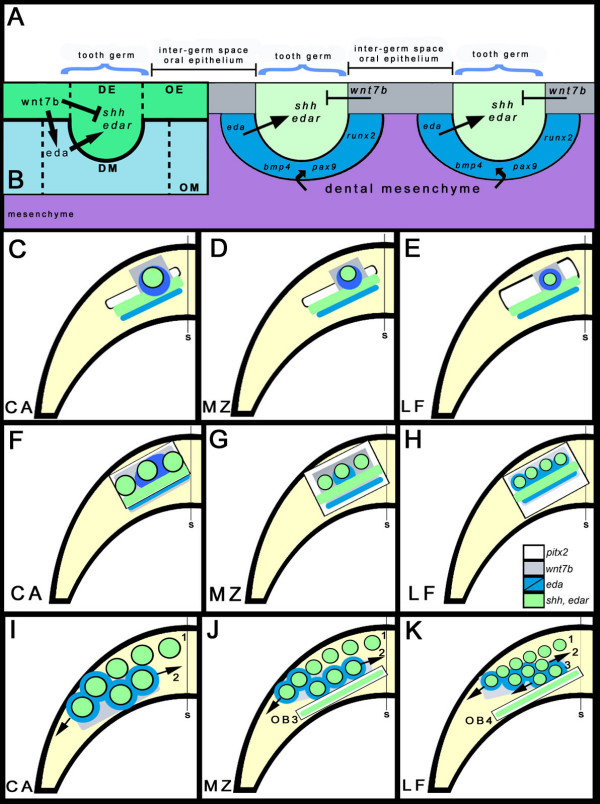
**A periodic pattern generator for diversity in Malawi cichlid dentitions**. (A) A cross-section through three thickened tooth germs showing planar signalling in the zone of inhibition (ZOI)/inter-germ space (grey, *wnt7b*) demarcating the location of teeth (green, *shh *and *edar*), with contribution from dental mesenchyme (blue, *eda*, *bmp4*, *pax9 *and *runx2*). Non-dental mesenchyme, purple. (B) The proposed interactions of wnt7b and eda (around the thickened tooth germs) with shh and edar within the thickened epithelial germ [9,30,44-51] in the context of our observations. wnt7b acts as an inhibitor of *shh *during tooth patterning and eda from the mesenchyme acts as an activator maintaining *shh*, perhaps via the eda-receptor *edar*, within the tooth germ. Wnt signals (possibly including wnt7b) may induce *eda *in the mesenchyme. DE, dental epithelium; DM, dental mesenchyme; OE, oral epithelium; OM, oral mesenchyme. (C)-(K) the induction of each tooth row acts in a 'copy-and-paste' mechanism utilizing the same genes for the pattern and organization of teeth and new tooth rows. Differential regulation of the genes during periodic patterning lead to the species diversityRedeployment of genes from OB1 establishes new tooth rows periodically in a lingual progression until the 'copy-and-paste' mechanism fails, due lack of combinatorial *pitx2 *and *shh*, essential for sequential row addition. Upper panel (C)-(E) around 5–6 days post-fertilization (dpf); middle panel (F)-(H) around 6–7 dpf; lower panel (I)-(K) around 12 dpf; each panel represents the left half of the dentary in dorsal view. (C)-(E the first tooth initiates from OB, a combinatorial expression of *pitx2 *(white) and *shh *(green). The initial *pitx2 *positive region is greatest in LF, with more rows. Up-regulation of *shh *and *edar *in the epithelial germs plus the establishment of a ZOI (*eda *and *wnt7b*) set the size and space restriction for the epithelial tooth germs. Local *eda *surrounds the first germ for each species (darker blue denotes stronger local expression of *eda*, which later becomes a broader domain and less intense, lighter blue). (F)-(H) *eda *remains strong and local in CA and at this stage (3–4-teeth stage) is broader and less intense in LF. CA does not initiate more than two tooth rows because *pitx2 *is constrained along the oral-aboral axis (lingual to the first teeth). The size, spacing and positioning of teeth and tooth rows is regulated by the interactions of *wnt7b *(grey), *eda *(blue) and *shh *(green) within the *pitx2*-positive field (white; see the main text and Figure 5). Future tooth row initiation in (J) MZ (OB3) and (K) LF (OB4) depends on the lingual co-expression of *pitx2 *and *shh*. (I) CA does not initiate a third row of teeth, and lacks co-expression of *shh *and *pitx2 *in a third OB (see Figure 4). Arrows mark the direction of tooth initiation for continued tooth addition on older rows. (I)-(K) *eda *and *wnt7b *expression domains are predicted from earlier stages. A more diffuse expression of *eda *(light blue) will continue to regulate size, spacing and row initiation of first-generation teeth. Numbers indicate the order of appearance for tooth rows. S, jaw symphysis.

Our data suggest that the ZOI has an important role in patterning the size (and spacing) of the *shh*-positive tooth germs, especially the first unit to initiate. We speculated that the first tooth might possess unique regulatory properties as a source for continued induction and patterning of the dental program. We tested the role of the first tooth as a source of communicative signal for the organization of the dentition using targeted chemical inhibition of the hedgehog pathway at the first epithelial thickening stage. *C. afra *embryos at the first-tooth stage (five days post-fertilization (dpf); Figure 2c) were treated for 24 hours in 50 μM cyclopamine in 1% DMSO (based on protocols in [52]), thus spanning the time from the initiation of the first tooth to the three-teeth stage (6 dpf), by which time the second row OB had established territory. A subset of treated embryos further developed for an additional 24 hours under standard conditions (7 dpf; Figure 7a and 7b); the remaining embryos from the same brood were allowed to develop for an additional six days (12 dpf; Figure 7c and 7d) to span the period of both first row eruption and development of the lingual tooth row (Figures 4, 7 and 8). Treated *C. afra *fixed at 7 dpf showed varying low levels of *shh *expression localized to a reduced number (one or two) of tooth germs on each side of the dentary and the OB for the second row (Figure 7b). *shh *expression appears within the region allocated for the ZOI (seen as *shh*-negative inter-germ regions in all controls; Figures 2c and 7a) normally expressing *eda and wnt7b *(Figures 5 and 6), suggesting a breakdown in both the initiation and spacing mechanisms (Figure 7b). Expression of *eda *in treated *C. afra *was absent in regions of tooth development (data not shown), implying that *eda *may respond to signals from, or downstream of, the hedgehog pathway. 1% DMSO control *C. afra *showed patterns of *shh *expression identical to standard controls (untreated).

In all treated individuals that were allowed to develop for a further six days (fixed at 12 dpf), we found that the first tooth continues partial development and shows signs of mineralization, although it does not complete development or attachment (Figure 7d). With the exception of a mineralized remnant of the first tooth, all other teeth, adjacent to the first and in subsequent rows, failed to develop (Figure 7d). Knockdown of the hedgehog pathway at the 3–4-teeth stage resulted in a functional, patterned and replacing dental system (data not shown). These observations demonstrate that when perturbed (via the hedgehog pathway) at the first-tooth stage, the dental programme cannot recover, despite continued cycles of periodic patterning past this stage in untreated individuals.

### The periodic pattern generator for dental diversity

The comparison of gene expression across Malawi cichlid species with divergent dentitions suggests a simple model implicating *pitx2*, *eda *and *wnt7b*, and their interaction with *shh *and *edar*, as primary features of a periodic pattern generator for diversity in Lake Malawi cichlid dentitions (Figure 8). The model accounts for two aspects of dental patterning: how to put tooth rows in jaws, and how to put teeth in tooth rows. Our data suggest that the combination of *pitx2 *and *shh *is required for a competent field of tooth initiation (the OB, Figures 2, 3, 4 and 8). *M. zebra *and *L. fuelleborni *exhibit expanded expression of *pitx2 *lingually on the embryonic lower jaw; *C. afra *does not (Figure 2). *pitx2 *and *shh *are also co-expressed in each subsequent OB for *M. zebra *and *L. fuelleborni *(Figure 4); *C. afra *does not initiate a third OB. Therefore, the lack of lingual/oral co-expression of *pitx2 *and *shh *in *C. afra *(Figures 4, 8F and 8I) may account for the reduction in row number compared with the other species (Figures 4, 8G and 8J, H and 8K, respectively). The lack of combinatorial expression of *shh *and *pitx2 *in the oral region of zebrafish may partially explain the lack of teeth [38]. Here we show that this mechanism likely accounts for variation in tooth row number among Malawi cichlids. Thus, molecular mechanisms used to pattern the first row of teeth (the only row of teeth in mammals and most vertebrates) are redeployed as 'triggers' of dental competence and initiation in each subsequent row. We suggest that the initiation of new tooth rows follows a 'copy and paste' mechanism wherein the dental expression network is redeployed for each new tooth row. Therefore, our model posits that preceding tooth rows are required as a source of signal to initiate the next lingual row during sequential addition.

The combination of comparative gene expression data and perturbation of the hedgehog pathway suggests that the correct initiation and maintenance of the first-tooth germ, via activation of *shh*, is necessary for the periodically patterned dental programme in Malawi cichlids (Figure 8). Comparison of the cyclopamine phenotype at the first-tooth to the 3–4-teeth stages shows that disturbing the development of the first-tooth germ has an effect on the entire dentition, whereas disrupting the dentition at later stages results in a mildly reduced phenotype with additional teeth forming and completing development. We do not yet understand the molecular mechanisms (for example, decreased epithelial proliferation and/or increased cell death) of this severe dental phenotype at the first-tooth stage.

Our data imply that *eda *and *wnt7b*, expressed in the ZOI, regulate initial tooth germ size and position within rows, through interactions with *shh*; wnt7b inhibits the germ through planar epithelial signals (Figures 5 and 6D–F) and eda maintains the tooth germ (*shh *and *edar*) from within the surrounding mesenchyme (Figures 5 and 6A–C). The ZOI may not lie solely within the layers of the epithelium and we suggest that inhibitor/activator controls signal from within the underlying mesenchyme that envelops the thickened dental epithelium [34]. Once the periodic pattern is established, other molecules may act as inhibitors from within the developing tooth unit, for example bmp2, which is present both in the early epithelial thickening and within the dental papilla (mesenchyme) during maturation (Figure 3J–L), and bmp4, which is restricted to the dental papilla (data not shown; [36]).

The expression of *eda *in the mesenchyme surrounding the developing dental germs of cichlids (Figures 5 and 6A–C) is more similar to that deployed during the patterning of feather placodes and salivary primordia [9] than that observed in mammalian dentitions, where it is restricted to epithelium [44,46]. In our model, a large initial tooth germ in *C. afra *results from sustained local and intense *eda *expression on a comparatively similar inhibitory background of *wnt7b *(Figure 5a). The size of this tooth germ is reduced in *M. zebra *(Figure 5b) and *L. fuelleborni *(Figure 5c) because the *eda *expression broadens earlier (especially for *L. fuelleborni*), a heterochronic imbalance setting the stage for more, closely packed *shh*-positive tooth germs (Table 1 and Figure 8). Consistent with our results, transgenic mice (K14-*eda*) with increased levels of *Ectodysplasin *expression exhibit larger tooth germs [48,52]. Furthermore, Eda null mutant mice have reduced tooth germs [48,53-55]. However, in the mouse, effects of Eda on tooth size correlate positively with effects on tooth number; for example, higher levels of *Eda *lead to a single extra molar [49,52]. Our data and model point to an important distinction between overall levels of *eda *and its spatial expression over time. An earlier dispersion of *eda *expression after initiation of the first tooth (as in *L. fuelleborni*), rather than continued localized expression around that first-tooth germ (as in *C. afra*), may in fact lead to the production of more, smaller tooth germs (Figure 8).

The position of subsequent tooth rows is also specified in part by the expression of *wnt7b *and *eda *in our model. Mesenchymal *eda *plays a permissive role in the positioning of the lingual OB (Figures 5 and 6A). In *C. afra*, its expression is strongest medial to the first tooth, while in *M. zebra *and *L. fuelleborni *it appears more as a band along the mesiodistal axis (Figures 5 and 6A–C; also see the second row tooth positions in Figure 1). *wnt7b *also appears to demarcate the location of the second row, as its expression is either side of the *shh*-positive second OB (Figures 5 and 6D–F) and, in a similar iterative manner to the patterning of individual tooth units, *wnt7b *is restricted to the inter-row space (Figure 5a–c). Once the initiation of the primary dental pattern for each row is established, the essential nature of *shh *and genes that occupy the ZOI is lost; although they likely continue to be expressed during further morphogenesis (Figures 3 and 6), these molecules are probably no longer required for initiation of the secondary, replacement dentition [13].

## Conclusion

Periodically patterned phenotypes such as the dentitions of Lake Malawi cichlids present important exemplars for evolutionary developmental biology. The discipline has heretofore focused on the molecular basis of evolutionary novelty among distantly related organisms [35,56] or the genetic/transcriptional basis of discrete trait loss among closely related groups [51,57]. Trait elaboration (for example, bigger, longer, stronger [58,59]) is more difficult to study because phenotypes are subtler, but this remains the more common type of evolutionary change [37]. Dental diversity is an intermediate case; quantitative elaboration takes the form of gain or loss of discrete units. Our results support the general model that old genes, and entire developmental modules, are deployed anew to generate micro-evolutionary novelty in iterative structures.

## Methods

### Fish husbandry

Embryos and fry of three species of Lake Malawi cichlids (*C. afra*, *M. zebra *and *L. fuelleborni*) were raised to the required stage in a recirculating aquarium system at 28°C. Embryo ages (in dpf) were set after the identification of mouth brooding females (day 0). Embryos were then removed from the mouths of brooding females and, if required, were maintained for further development in separate culture tanks at 28°C.

### Sequences

Cloned sequences used to generate digoxigenin-labelled antisense riboprobes from Malawi cichlid species have been deposited in GenBank (accession numbers: EU867210 – EU867219). Many of the genes were identified through partial genome assemblies of *L. fuelleborni *and *M. zebra *[60] and cloned from *M. zebra *and *L. fuelleborni *cDNA libraries. Sequences of cDNA used to generate the probes are identical across the three species. Overall, these species exhibit almost no sequence divergence; the average nucleotide diversity for comparisons across the Malawi assemblage is 0.2%, less than among laboratory strains of the zebrafish [60].

### *In situ *hybridization

To ensure the embryos of the three species were of equivalent stages (especially during gene expression comparisons), specimens were stage-matched based on external features, including pectoral and caudal fin development and eye development and maturity. Specimens for *in situ *hybridization were anaesthetized in tricaine methanesulfonate (MS222, Argent) and fixed overnight in 4% paraformaldehyde (PFA) in 0.1% phosphate-buffered saline (PBS) at 4°C. Whole-mount *in situ *hybridization experiments were based on protocols from [12] and modified as follows: embryos were transferred to methanol for dehydration and stored at -20°C. Specimens were rehydrated through to PBS with Tween-20 and digested with 4–10 μg/ml proteinase K (PK); the final concentration was based on the specific stage of embryo/fry (for example, embryos at approximately 5 dpf were digested with 5 μg/ml PK). Following hybridization, embryos were washed in TST (10 mM NaCl, 10 mM Tris-HCl, Tween-20 in depc-H_2_O). During the colour reaction stage of the protocol, all embryos were allowed to fully develop the colour. Thus, embryos were continuously transferred into fresh NBT/BCIP solution (Roche) in NTMT until full staining had ensued; this was determined after multiple regions of known expression became positive. Specimens were stage-matched based on external features, including pectoral and caudal fin development and eye development and maturity. All *in situ *hybridization experiments were performed with multiple specimens (multiple individuals were fixed at regular intervals, within single broods, then repeated at least twice with alternative broods) to fully characterize the expression patterns within and across the three species. After colour reaction (NBT/BCIP, Roche) embryos were washed in PBS and fixed again in 4% PFA, before whole-mount imaging using a Leica Microsystems stereomicroscope (MZ16). Embryos were embedded in gelatin and chick albumin with 2.5% gluteraldehyde. The gelatin-albumin blocks were post-fixed in 4% PFA before sectioning. Thin sections were cut at 15–25 μm using a Leica Microsystems VT1000 vibratome.

### Cyclopamine manipulation of the hedgehog pathway

From a single brood of 24 individuals, 14 *C. afra *embryos were treated with cyclopamine (LC Laboratories) compound (50 μM) from a stock (5 mM cyclopamine in DMSO) to make up a final 1% DMSO solution in fish water. Five *C. afra *individuals were used as a 1% DMSO control, under the same incubation conditions as the treated embryos (Figure 7a and 7c). A further five individuals were kept as standard controls (wild-type), developing in the Georgia Institute of Technology aquarium. Treatment and control experiments were performed in ventilated Petri dishes spinning at 28°C in an oscillating platform culture incubator (Barnstead Lab-Line Max 4000). Following the treatment experiments and for the controls with DMSO, fishes were washed 10 times in fresh fish water to remove any remnant of cyclopamine compound or DMSO before transferring to culture vessels containing at least 300 ml of fish water, changed daily until ready for analysis. Although initial experiments with 50 μM cyclopamine using 1% (of 95%) ethanol as the solvent (suggested by the manufacturer, LC Laboratories and previous reports [51,61]) showed differential expression patterns of *shh *to the 1% ethanol control experiments, alizarin red preparation of embryos raised to 12 dpf showed gross phenotypic effects on the ethanol-administered controls. Therefore, we substituted 1% DMSO for ethanol solvent, after which controls could not be distinguished from standard controls (untreated). While DMSO is not the best solvent for cyclopamine because of limited solubility above concentrations of 4 mg/ml, at the low concentrations used for enhanced viability of treated embryos, DMSO proved to be a better solvent than ethanol because of lower solvation temperatures and faster solvation times from -20°C storage temperatures.

## Authors' contributions

GJF and JTS designed the study. GJF carried out the analyses. RFB performed the cyclopamine treatment experiments. All authors contributed to the preparation of the manuscript and read and approved the final version.
